# Transcriptome and Metabolome Analyses Reveal High-Altitude Adaptation in the Qinghai Toad-Headed Lizard *Phrynocephalus vlangalii*

**DOI:** 10.3390/biology14050459

**Published:** 2025-04-24

**Authors:** Jun Zhong, Jian Chen, Yu-Hong Lu, Yu-Fei Huang, Ming-Sheng Hong, Xiang Ji

**Affiliations:** 1 Zhejiang Provincial Key Laboratory for Water Environment and Marine Biological Resources Protection, College of Life and Environmental Sciences, Wenzhou University, Wenzhou 325035, China; zhongjun@wzu.edu.cn (J.Z.); chenjian20233@163.cm (J.C.); luyuhongehh@outlook.com (Y.-H.L.); hyf578829865@outlook.com (Y.-F.H.); 23211270148@stu.wzu.edu.cn (M.-S.H.); 2 Institute for Eco-Environmental Research of Sanyang Wetland, Wenzhou University, Wenzhou 325014, China

**Keywords:** carbohydrate metabolism, fatty acid metabolism, glycolysis, high altitude, *Phrynocephalus vlangalii*

## Abstract

The plateau environments are always harsh, with low pressure, low oxygen, and low temperature, which are detrimental to the survival of organisms. The Qinghai toad-headed lizard *Phrynocephalus vlangalii* has a wide range of altitude adaptation from 2000 to 4600 m. But it is still unclear how organisms maintain tissue function by balancing energy supply and demand changes in high-altitude environments of *P. vlangalii*. We investigated the plateau metabolic adaptation through transcriptome and metabolome analyses of *P. vlangalii* from three populations at different altitudes of the Qinghai-Tibet Plateau. The genes related to carbohydrate metabolism were significantly down-regulated at the high altitude. The metabolites alpha-D-glucose 1-phosphate, beta-D-fructose 6-phosphate, D-glycerate 1,3-diphosphate, 3-phosphoglycerate, and phosphoenolpyruvate in glycolysis/gluconeogenesis were down-regulated, too. The lipid metabolic and fatty acid synthase-related genes were up-regulated at a high altitude. In conclusion, the glycogen utilization-related genes and metabolites experienced broad down-regulation, while lipid-related genes and metabolites had a clear trend of up-regulation. Thus, we suggest that *P. vlangalii* tends to increase lipid utilization and reduce the dependence on glycogen consumption to acclimatize to the high-altitude environment.

## Simple Summary

The adaptation of organisms to high altitudes has always been a key concern. Here, we investigated high-altitude adaptation in the Qinghai-Tibet Plateau lizard *Phrynocephalus vlangalii*. The lizard showed an increased resting metabolic rate with altitude, tending to increase lipid utilization and reduce dependence on glycogen consumption to adapt to the high-altitude environment. Our study provides new insights into the metabolic plasticity of reptiles and complements existing research on high-altitude adaptations.

## 1. Introduction

Ascent to a high altitude places organisms in an adverse environment and exposes them to multiple stressors, reduced barometric pressure (and thus reduced oxygen partial pressure, hypoxia), low temperature, and increased ultraviolet radiation [1], of which the negative biological effects include oxidative stress, metabolic disorders, and UV damage [2,3,4]. Organisms cannot live at high altitudes without physiological adjustments [5,6,7]. Metabolism as an important physiological activity, forming a bridge for signal sensing and transmission between organisms and the external environment. Animals systematically or temporarily change their metabolites when they are affected by external factors, and this is studied in research on topics such as the diet, living habits, environment, genetic effects, and drugs [8,9,10].

The resting metabolic rate (RMR) is a measure of the energy required by animals at rest [11]. Many animals can alter their RMR to adapt to different conditions. In the tegu lizard *Tupinambis merianae*, for instance, the RMR varies seasonally, with a higher RMR in higher-activity seasons than in low-activity seasons even at the same temperature [12]. For animals living at a high altitude, resisting the cold through aerobic heat production is necessary for survival, while metabolic inhibition to reduce the oxygen demand is not feasible [13,14,15]. For instance, high-altitude sheep (*Ovis ammon*) have an increased hemoglobin-O2 affinity compared with their low-altitude conspecifics [16]. Usually, they alter the activity of regulatory enzymes across an altitudinal gradient [17,18,19,20]. For instance, increased fatty acid storage and metabolism appear to be common responses among animals at high altitudes [7,13,21,22].

There are three key enzymes in the major energy-generating pathways. β-hydroxyacyl-CoA dehydrogenase (HOAD) catalyzes the β-oxidation of fatty acids [23]. Citrate synthase (CS) catalyzes the first and rate-limiting reaction of the citric acid cycle [23]. Lactate dehydrogenase (LDH) catalyzes the conversion of pyruvate, the end product of glycolysis, into lactate [23]. In diving pinnipeds seals (*Phoca vitulina*) and sea lions (*Eumetopias jubatus*), HOAD and CS activities are elevated to maintain an aerobic, fat-based metabolism [24]. In oxygen-supplemented rats (*Rattus rattus* and *R. norvegicus*), the lactate dehydrogenase levels increased rapidly [7,25]. The high-altitude Andean goose (*Chloephaga melanoptera*) and puna teal (*Spatula puna*) exhibited increases in HOAD activity and decreases in LDH activity [26]. Yaks (*Bos grunniens*) from high altitudes show higher activities of HOAD and LDH [27]. Yaks increase collagen synthesis to maintain alveolar stability at low altitudes, and they decrease the collagen fiber content in the lung tissues at high altitudes [28]. In high-altitude populations of *Bufo gargarizans* and *Rana kukunoris*, genes related to material metabolism are positively selected [29]. Yarkand toad-headed lizards (*Phrynocephalus axillaris*) and Forsyth’s toad-headed lizards (*Phrynocephalus forsythii*) reduce the behavioral intensity and increase energy efficiency when transferred to high-altitude environments [20].

Research about model prediction reveals that with global warming, animals will migrate to higher altitudes [2,3,4,30]. Therefore, adaptation of animals to high altitudes is becoming particularly important. Laboratory studies controlling a single variable cannot effectively explain adaptation to high altitudes, such as chronic hypoxia, or low temperatures [31,32,33]. However, the actual plateau environment may be more complex than a single-experimental-variable environment, and some questions have not been fully answered. Qinghai toad-headed lizards (*Phrynocephalus vlangalii*) live on the Tibetan Plateau across an altitudinal range from 2000 to 4600 m [34]. In this study, we collected livers from adult males of *P. vlangalii* from three altitudes, and we performed transcriptome and metabolome analyses, aiming to uncover the molecular mechanism for altitude adaptation in *P. vlangalii*.

## 2. Materials and Methods

### 2.1. Animal Collection and Treatment

We collected adult male lizards in July 2020 from three populations, one [Aksay (AKS), 94.35° N, 38.80° E, 2500 m altitude] in Gansu Province and two [Nachitai (NCT, 94.90° N, 36.40° E, 3500 m altitude) and Xidatan (XDT, 94.17° N, 35.72° E, 4200 m altitude)], in Qinghai Province. Forty-three lizards were transferred to our laboratory, where 4−6 individuals were housed in each 800 × 600 × 500 mm (length × width × height) plastic cage with a sand substrate (120−150 mm depth). Cages holding lizards from different populations were placed in a room where temperatures varied from 24 °C to 28 °C and lights were on a 12:12 light–dark cycle. Lizards were provided with a sufficient supply of mealworms (larvae of *Tenebrio molitor*) and water.

### 2.2. Resting Metabolic Rate (RMR) Measurement and Data Analysis

Ten adult males larger than 50 mm SVL from each population were used to measure RMR at 28 °C in August (Table S1). Before RMR measurement, lizards were fasted for 24 h, and then they were individually placed in a 250 mm long cylinder with an inner diameter of 20 mm, and maintained at a flow rate of 600 mL min^−1^. The air was dried in a drying column (Driecite™, Morristown, NJ, USA), then the dried air was passed through a closed-flow O_2_/CO_2_ analysis system (FOXBOX-C, Sable Systems, North Las Vegas, NV, USA) to measure the O_2_ and CO_2_ concentration variation [35]. The experiment was conducted between 7:00 and 17:00 h. During the measurement of RMR, the experimental temperature was controlled by a constant-temperature system at 28 °C.

Prior to parametric analyses, data on RMR were tested for normality (Kolmogorov–Smirnov test) and homogeneity of variances (Bartlett’s test) in STATISTICA 10.0 (Stat Soft, Tulsa, OK, USA). We used one-way ANCOVA (with body mass as the covariate) to test whether RMRs differed among the three populations and, if so, we further performed Tukey’s post hoc multiple comparisons to show where the difference(s) lied. Descriptive statistics were shown as the mean ± standard error, and the significance level was set at 0.05.

### 2.3. Transcriptome Analysis

Lizards were fasted for 10 h at the site of collection before they were killed by decapitation to collect the whole liver, which was divided into two pieces (one for transcriptome analysis and one for metabolome analysis) and then quickly frozen in liquid nitrogen. Total RNA was extracted using a Trizol reagent kit (Invitrogen, Carlsbad, CA, USA) according to the protocol. After testing for completeness and purity of total RNA, mRNA was enriched by Oligo(dT) beads (New England Biolabs, Ipswich, MA, USA). The mRNA was fragmented into short fragments and reverse transcribed into cDNA with random primers. The cDNA fragments were purified with a QiaQuick PCR extraction kit (Qiagen, Venlo, The Netherlands), end repaired, poly(A) added, adapters ligated, PCR amplified, etc., and the products were sequenced using Illumina HiSeqTM 4000 by Gene Denovo Biotechnology Co. (Guangzhou, China). Three biological replicates were detected for each altitude [27].

Raw reads were filtered by fastp 0.18.0 to obtain high-quality clean reads for future study [36]. Transcriptome de novo assembly was carried out through Trinity [36]. The unigene expression was calculated and normalized to FPKM (fragment per kilobase of transcript per million mapped reads) [37]. We used the BLASTx program (http://www.ncbi.nlm.nih.gov/BLAST/; accessed on 20 June 2021) with an E-value threshold of 1 × 10^−5^ to the NCBI non-redundant protein (Nr) database (http://www.ncbi.nlm.nih.gov; accessed on 20 June 2021), the Swiss-Prot protein database (http://www.expasy.ch/sprot; accessed on 20 June 2021), the Kyoto Encyclopedia of Genes and Genomes (KEGG) database (http://www.genome.jp/kegg; accessed on 20 June 2022), and the COG/KOG database (http://www.ncbi.nlm.nih.gov/COG; accessed on 20 October 2020) to annotation the unigenes. Differentially expressed genes (DEGs) were examined by DESeq2 [38] between two groups. The genes with a false discovery rate (FDR) below 0.05 and absolute fold change ≥ 2 were considered DEGs. The GO enrichment analysis of DEGs was conducted in the Gene Ontology database (http://www.geneontology.org/; accessed on 28 July 2021), and significantly enriched GO terms were defined by the hypergeometric test with a *p*-value < 0.05. Fisher’s exact test method was used to perform KEGG pathway enrichment analysis on DEGs according to the Kyoto Encyclopedia of Gene and Genomes (https://www.kegg.jp/; accessed on 28 July 2021) database, and when *p* < 0.05, it was considered a significantly enriched pathway.

### 2.4. Metabolome Analysis

The metabolites were subjected to methanol extraction and analyzed by a Liquid Chromatograph Mass Spectrometer (Thermo Ultimate 3000, Waltham, Germany) [39]. Simultaneously, two ionization methods, positive-ion-mode metabolites (POS_MS) and negative-ion-mode metabolites (NEG_MS), were detected together to increase the coverage of metabolites and improve the detection effect. Six biological replicates were detected for each altitude. The raw dates were converted into the mzXML format by Proteowizard software (v3.0.8789). The package XCMS in R 3.3.2 was used for peak identification, filtering, and alignment [40]. The peak intensities were batch-normalized to the total spectral intensity. The mass spectrometry information was matched with the public metabolic database Human Metabolome Database (http://www.hmdb.ca; accessed on 15 May 2021), METLIN (http://metlin.scripps.edu; accessed on 15 May 2021), Massbank (http://www.massbank.jp/; accessed on 15 May 2021), LipidMaps (http://www.lipidmaps.org; accessed on 15 May 2021), and mzClound (https://www.mzcloud.org; accessed on 15 May 2021) to confirm the annotations for metabolites, and the peaks that had not been compared were removed from subsequent analysis.

The orthogonal projection-discrimination analysis (OPLS-DA) was used to distinguish differential metabolites (DAMs) between groups. By combining the variable importance projection (VIP) value from OPLS-DA with the P of a univariate statistical analysis *t*-test, we screened the significant DAMs with a VIP ≥ 1 and *p* < 0.05. Fisher’s exact test method was used to perform KEGG pathway enrichment analysis on DAMs, and the significance was considered with *p* < 0.05.

### 2.5. Association Analysis of Metabolome and Transcriptome

We used a correlation coefficient model to calculate the Pearson correlation coefficient (r) between differentially expressed genes and differential metabolites. The screening criteria were *p* < 0.01 and absolute r > 0.9. The top genes and metabolites were selected for heatmap analysis using pheatmap packages in R project. We mapped the DEGs and DAMs involved in the same KEGG pathway to analyze and reveal the metabolism difference between altitudes.

## 3. Results

### 3.1. Altitudinal Trend of Resting Metabolic Rate (RMR)

**Figure 1:**
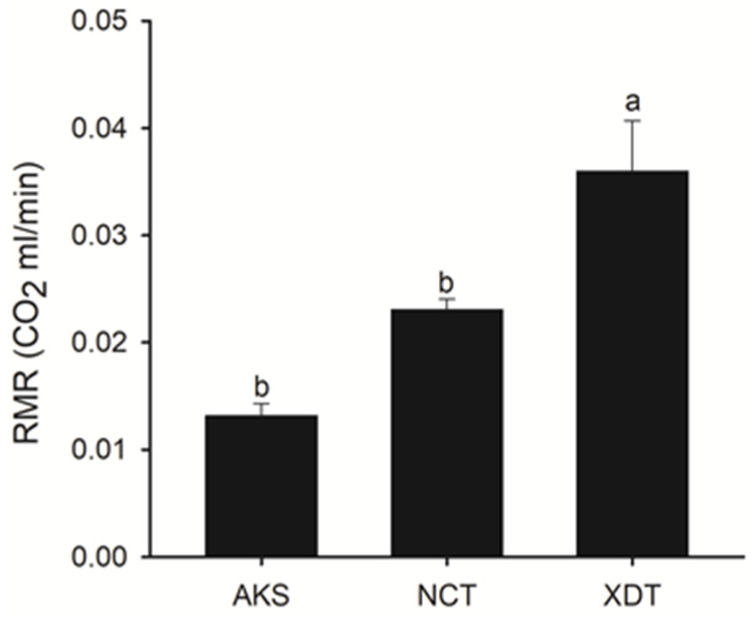
Adjusted means (+SE) for resting metabolic rate (RMR) of *Phrynocephalus vlangalii* from three altitudes, with body weight set at 6.928 g. Adjusted means with different letters differ significantly (Tukey’s test, a > b). The number of samples was 10 at each altitude. AKS: Aksay; NCT: Nachitai; XDT: Xidatan.

The RMR differed significantly among the three populations (ANCOVA; F_2,26_ = 15.472, *p* < 0.001). The RMR was significantly greater in the XDT population than in another two populations after accounting for body mass, with the mass-adjusted mean RMR being greatest in the XDT population (highest) and smallest in the AKS population (lowest) (Figure 1).

### 3.2. Differently Expressed Genes (DEGs) Revealed the Metabolism Change with Altitude

A total of 82,438 unigenes with an average length of 886 bp were obtained from the transcriptome databases. The N50 length was 1705 bp, and the N50 number was 11,192. A total of 35,615 genes were annotated in at least one database, 18,984 genes were annotated in all four databases, and 13,125 orthologous transcripts were identified in Pogona vitticeps (Table S2).

**Figure 2:**
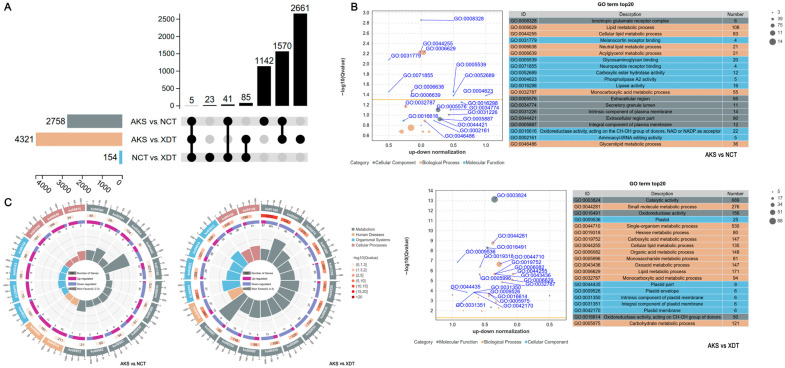
Global transcriptomic profiles of *P. vlangalii* significantly change with altitude. (**A**) Differentially expressed genes (DEGs) among different altitudes in *P. vlangalii*. (**B**) GO enrichment analysis of DEGs in the AKS versus NCT (**up**) population and the AKS versus XDT (**down**) population. (**C**) KEGG enrichment analysis of DEGs in the AKS versus NCT (**left**) population and the AKS versus XDT (**right**) population.

Using the AKS population as a reference, we found 2758 DEGs (1347 up-regulated and 1411 down-regulated) in the NCT population and 4321 DEGs (1456 up-regulated and 2865 down-regulated) in the XDT population; fewer DEGs were found in the XDT population (58 up-regulated and 96 down-regulated), as compared with the NCT population (Figure 2A). The trend analysis showed that the expression patterns of 12,852 unigenes were similar in the XDT and NCT populations (Figure S1). The expression of 4847 genes differed with altitude, with 3353 down-regulated and 1494 up-regulated (Figure S1; Table S3). Among them, 1570 genes had significant expression pattern change in the NCT and XDT populations when compared with the AKS population; 625 were co-up-regulated, and 945 were co-down-regulated (Figure 2A). For example, lactate dehydrogenase encoding gene LDHA (Unigene0035661) and phosphoglycerate kinase encoding gene PGK1 (unigene0025643) were co-down-regulated (Table S3). The expression of glutathione S-transferase A4-like encoding gene Gsta4 (Unigene0063515) was significantly up-regulated with altitude, which indicated that as the altitude increases, the detoxification ability needs to be enhanced (Figure 2A; Table S3). The expression of pyruvate dehydrogenase kinase encoding gene PDK was significantly increased in lizards from the NCT (PDK3, Unigene0071827) and XDT (PDK4, Unigene0024469) populations (Figure 2A; Table S3).

The GO analysis indicated that 565 DEGs (280 up-regulated and 285 down-regulated) in the NCT population and 946 DEGs (355 up-regulated and 591 down-regulated) in the XDT population were associated with the metabolic process, as compared with the AKS population. In addition, compared with the AKS population, the lipid metabolism-related pathway was significantly enriched in the NCT and XDT populations, such as cellular lipid metabolism (GO:0044255), neutral lipid metabolism (GO:0006638), acylglycerol metabolism (GO:0006639), and glycerolipid metabolism (GO:0046486) (Figure 2B and Figure S2). We found that 108 DEGs in the NCT population were associated with lipid metabolism (GO:0006629), and nearly a half were up-regulated; 13 genes related to lipase activity (GO:0016298) were up-regulated; and the expression of 9 genes participating in carboxylic ester hydrolase activity (GO:0052689) was enhanced in the NCT population, as compared with the AKS population (Figure 2B and Figure S2). In the XDT population, 121 DEGs participated in carbohydrate metabolism (GO:0005975). Additionally, hexose metabolism (GO:0019318) and monosaccharide metabolism (GO:0005996) were significantly enriched (Figure 2B and Figure S2). Over 70% (85/121) of DEGs involved in carbohydrate metabolism were down-regulated, and the plastid components were also weakened in the XDT population (Figure S2). Additionally, in the XDT population, lipid metabolism was significantly enriched, too, and 79 genes were up-regulated (Figure S2).

Through KEGG analysis, a total of 330 and 346 KEGG pathways were found in the NCT and XDT populations, respectively, when compared with the AKS population. Among these pathways, 328 were found both in the NCT and XDT populations, and 93 were related to metabolism (Table S4). In the NCT population, 55, 34, and 48 DEGs participated in carbohydrate, amino acid, and lipid metabolism, respectively. The lipid metabolism-related pathway, arachidonic acid metabolism (ko00590), linoleic acid metabolism (ko00591), and glycerophospholipid metabolism (ko00564) most significantly differed, with more up-regulated DEGs (Figure 2C, Table S4). In the XDT population, 150, 81, and 81 DEGs were involved in carbohydrate, amino acid, and lipid metabolism, respectively. The carbohydrate-related pathway, fructose and mannose metabolism (ko00051), glycolysis and gluconeogenesis (ko00010), and carbon metabolism (ko01200) most significantly differed, with more down-regulated DEGs (Figure 2C, Table S4).

### 3.3. Metabolomic Data Revealed the Glycogen and Lipid Metabolism Change with Altitude

We detected 13,218 metabolites (MS) (6248 POS_MS and 7010 NEG_MS) and annotated 2158 MS (1228 POS_MS and 932 NEG_MS) through comparison with the database (Table S5). Lipids were most diverse (14.3%), mainly including 94 steroids, 63 prenol lipids, and 142 fatty acyls, followed by organic acids and derivatives (13.2%) and organic oxygen compounds (7.7%) (Table S5). Organic acids and derivatives mainly included 210 amino acids, peptides, and analogs, and organic oxygen mainly divided into three categories, carbonyl compounds, carbohydrates and carbohydrate conjugates, and alcohols and polyols (Table S5).

**Figure 3:**
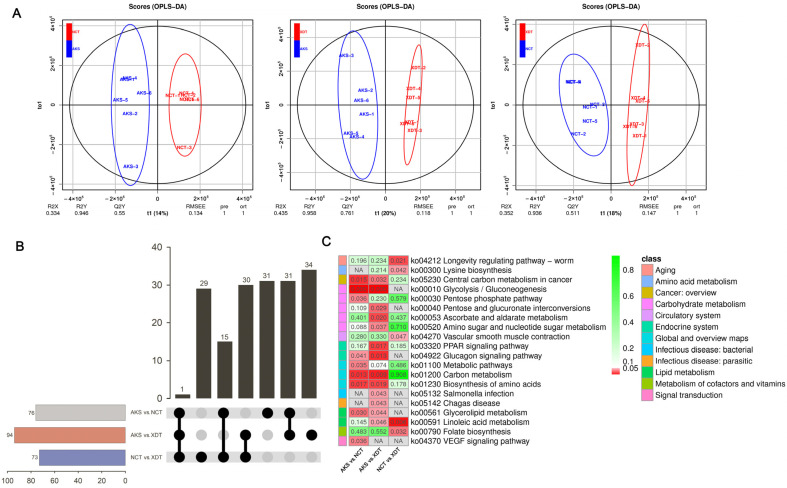
Global metabolomic profiles of *P. vlangalii* show a significant change with altitude. (**A**) OPLS-DA scores show significant differences of AKS versus NCT (**left**), AKS versus XDT (**middle**), and NCT versus XDT (**right**). (**B**) Differential metabolites (DAMs) among different altitudes in *P*. *vlangalii*. (**C**) KEGG enrichment analysis of DAMs. The bar from red to green shows *p* values from low to high.

The OPLS-DA showed that both metabolites of the XDT population and the NCT population were significantly separated from the AKS population (Figure 3A). Based on OPLS-DA models, we observed 76 DAMs (33 up-regulated and 43 down-regulated) in the NCT population, and 94 (49 up-regulated and 45 down-regulated) in the XDT population, when compared with the AKS population (Figure 3B, Table S6). DAMs were identified in amino acid metabolism, carbohydrate metabolism, and lipid metabolism, with 14, 13, and 10 in the NCT population, and 19, 12, and 8 in the XDT population, respectively (Figure S3). Thirty-one DAMs were both found in the NCT and XDT populations (Figure 3A). Twenty-one MS were co-down-regulated in the NCT and XDT populations. The most co-down-regulated DAMs were carboxylic acids and derivatives (10/21) such as isocitric aci (POS_M192T115), N-acetylhistidine (POS_M198T99), and glycylleucine (NEG_M187T192). These were followed by fatty acyls (5/21) and five organooxygen compounds (5/21), which included octadecanamide (POS_M284T502), 9,10-DHOME (NEG_M313T747), D-glucose 1-phosphate (NEG_M259T77), galactaric acid (NEG_M209T76), and rimantadine (POS_M162T431) (Tables S5 and S6). Nine MS were co-up-regulated, including two carboxylic acids and derivatives, N-Acetylhistidine (POS_M198T99), 1-Methylhistidine (POS_M170T93), and one carbohydrate and carbohydrate conjugate beta-Lactose (POS_M360T100) (Tables S5 and S6). Cyanidin 3-glucoside (NEG_M449T90) was down-regulated in the NCT population while it was up-regulated in the XDT population.

The most important metabolic variation was observed in glycolysis/gluconeogenesis (ko00010) with altitude. MS involved in glycolysis/gluconeogenesis was decreased in the NCT and XDT populations when compared to the AKS population, such as alpha-D-glucose 1-phosphate and beta-D-fructose 6-phosphate (Figure 3C; Table S7). Additionally, carbon metabolism (ko01200), glycerolipid metabolism (ko00561), glucagon signaling pathway (ko04922), and biosynthesis of an amino acid (ko01230) were significantly changed in the NCT and XDT populations (Figure 3C; Table S7), and linoleic acid metabolism (ko00591) and pentose and glucuronate interconversions (ko00040) were significantly changed in the XDT population (Figure 3C; Table S7).

### 3.4. Joint Analysis of Transcriptome and Metabolome Changes

**Figure 4:**
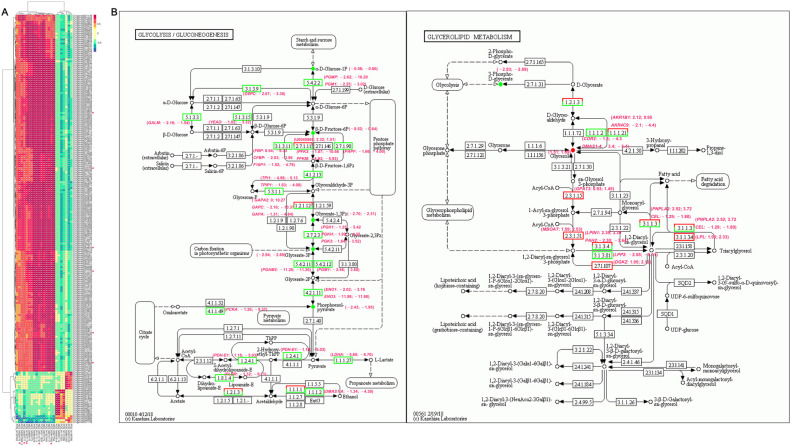
Integrated enrichment analysis of differential metabolites (DAMs) and differently expressed genes (DEGs). (**A**) Heatmap of the top DAMs and DEGs. The color of the squares in the graph represents a correlation, with darker colors indicating a stronger correlation. *, *p* < 0.05. (**B**) KEGG pathway enrichment of DAMs and DEGs and pathway detail, glycolysis/gluconeogenesis (**left**) and glycerolipid metabolism (**right**). The green dots and rectangle represent down-regulation, the red ones represent up-regulation. The numbers in parentheses represent the log_2_ (fold change) of gene expression or metabolism accumulation in the NCT (**left**) and XDT populations (**right**) as compared to the AKS population.

A total of 159 DAMs were significantly correlated with 6037 DEGs at *p* < 0.01 and |*r*| > 0.9 among the AKS, NCT, and XDT populations, resulting in 20,214 related pairs (2090 negative, 18,124 positive) (Table S8). The heatmap displayed the top correlation between DEGs and 23 DAMs. A lot of genes showed a positive relation with D-glucose 1-phosphate, galactaric acid, isocitric acid (POS_M192T115), rimantadine (POS_M162T431), octadecanamide, and 9,10-DHOME (Figure 4A). Certain genes with important functions, such as Serhl (serine hydrolase-like protein 2, Unigene0064820), Ak3 (phosphotransferase AK3, Unigene0076291), TCAF2 (TRPM8 channel-associated factor, Unigene0032729), TCAF (Unigene0032730), TMEM272 (transmembrane protein 272, Unigene0011125), and HSP30 (heat shock protein 30C-like, Unigene0024956), were positive related to these MS with altitude (Figure 4A). Additionally, 15 fatty acyls were correlated with 2075 DEGs, and the prostaglandin I2 (POS_M352T835) was correlated with LDH (unigene0035661) (Table S5). EHHADH (unigene0007755) was positively correlated with galactaric acid (NEG_M209T76), and CS (unigene0037876) was positively correlated with 6-aminopenicillanic acid (POS_M217T133) (Table S6). The glycogen synthase-related gene GYS (unigene0075253) was significantly negatively correlated with L-arginine (POS_M175T92) and positively correlated with steroid emtricitabine (NEG_M247T91) (Table S6).

The KEGG pathway map linking genes and metabolites showed that the glycolysis/gluconeogenesis process negatively correlated with altitude. The expression of ALDOA (unigene0058703; fructose-bisphosphate aldolase A), PGAM2 (unigene0010537; phosphoglycerate mutase 2), PGM1 (unigene0039628; unigene0019279; phosphoglycerate mutase 1), PDH (unigene0010107; pyruvate dehydrogenase), PGK1, PGKH (unigene0019715; phosphoglycerate kinase), PGK2 (unigene0022532), and LDHA decreased by more than two times at the higher altitude (Figure 4B, Table S2). Five MS were down-regulated with altitude, including alpha-D-glucose 1-phosphate, beta-D-fructose 6-phosphate, D-glycerate 1,3-diphosphate, 3-phosphoglycerate, and phosphoenolpyruvate (Figure 4B). Additionally, the FoxO signaling pathway was enhanced in the XDT population, and the transcription factor FoxO1 (unigene0077053; forkhead box protein O1) was up-regulated in the NCT and XDT populations (Figure S4A; Table S3).

The glycerolipid metabolism process was positively correlated with altitude. The expression levels of MBOAT2 (unigene0022312; lysophospholipid acyltransferase 2), GPAT3 (unigene0044507; glycerol-3-phosphate acyltransferase 3), LPIN1 (unigene0001893; phosphatidate phosphatase), and LPL (unigene0019390; lipoprotein lipase) were up-regulated with the altitude (Figure 4B, Table S3). The metabolite glycerol significantly increased at a high altitude (Figure 4B, Table S3). The up-regulated PPAR γ (unigene0077608; peroxisome proliferator-activated receptor gamma) promoted the expression of fatty acid transport and oxidation-related genes in *P. vlangalii* from high-altitude populations. The expression of ACSL5 (unigene0075828; acyl-CoA synthetase enzymes), FABP (unigene0074047; fatty acid-binding protein), ACOX2 (unigene0006923; peroxisomal acyl-coenzyme A oxidase 2), LPL (unigene0002145), and ketone synthesis gene HMGCS2 (unigene0018787; hydroxymethylglutaryl-CoA synthase) was up-regulated in the NCT and XDT populations (Figure S4B; Table S3). The expression of fatty acid metabolic-crucial genes SREBF1 (unigene0061775; sterol regulatory element-binding transcription factor 1), FASN (unigene0031318, fatty acid synthase), ACADVL (unigene0059287; very long-chain-specific acyl-CoA dehydrogenase), and MECR (unigene0077910, trans-2-enoyl-CoA reductase) was up-regulated, too (Table S3).

## 4. Discussion

When transplanted to a high-altitude site, two low-altitude species, *Phrynocephalus axillaris* and *P. forsythia,* reduce the tail display behavior and increase energy efficiency to acclimate to high-altitude environments [20]. In this study, we found that *P. vlangalii* tended to increase the RMR at high altitudes. The high-altitude *P. vlangalii* decreased glycogen utilization and increased lipid metabolism to adapt to the environment. The metabolomic and transcriptomic profiles showed that along the altitudinal gradient, glycogen utilization-related process were down-regulated, while the lipid-related process was up-regulated.

Increasing metabolism to maintain energy homeostasis in response to a low temperature and high-altitude pressure is very common in animals [41,42,43,44,45]. Plateau pikas (*Ochotona curzoniae*) have an unusually high RMR as an auxiliary way to adapt to the extreme cold of the Qinghai-Tibet Plateau [41]. In the Mesquite lizard (*Sceloporus grammicus*), the RMR is higher in a high-altitude population than in a low-altitude one [45]. Similarly, we found that the RMR of *P. vlangalii* from high-altitude populations increased. In fact, insects rarely occur at altitudes higher than 4000 m [46]. The lack of a sufficient energy and fat supply due to the insect scarcity may make it difficult to migrate to higher altitudes for *P. vlangalii*.

As the primary energy storage materials for animals, glucose and lipid play a key role in high-altitude adaptation. However, different animals have different ways of managing energy utilization to adapt to high-altitude environments. In high-altitude deer mice (*Peromyscus maniculatus*), fatty acid metabolism is enhanced, whereas the TCA cycle and glycolysis remain unchanged [13,22,47]. When mice (*Mus musculus*) are treated with a simulated altitude of 5000 for a short time, glycolysis is weakened by significantly decreasing the activities of key glycolytic enzymes [48]. In gayals (*Bos frontalis*) from the alpine canyon and yaks from the Qinghai-Tibet Plateau, energy metabolism shifts from fatty acid degradation to glycolysis, and the LDH activity is positively correlated with altitude [21,49,50]. Heterotherms and homeotherms differ in high-altitude adaptation. Asiatic toads (*Bufo gargarizans*) adapt to high altitudes by reducing nutrient metabolism [28,51,52]. Two low-altitude toad-headed lizards, *P. axillaris* and *P. forsythii*, increase energy efficiency by increasing the expression of SREBF1 and FASN, when transplanted to high altitudes [20]. As in *P. axillaris* and *P. forsythii*, SREBF1 and FASN were up-regulated at high altitudes in *P. vlangalii*, indicating that there may be some commonalities among reptiles in high-altitude adaptation.

In our study, several core genes and metabolites associated with glycogen utilization were down-regulated in *P. vlangalii* from high-altitude populations. The metabolic 3-phosphoglycerate is an important intermediate metabolite in glycolysis/gluconeogenesis; it is the product of the reduction of 1,3-diphosphoglycerate from glucose conversion. Then, 3-phosphoglycerate converts to 2-phosphoglycerate and enters the next step of glucose oxidation. PGM catalyzes the reversible interconversion of 3-phosphoglycerate and 2-phosphoglycerate in the glycolytic pathway [53]. PGK catalyzes 1,3-biphosphoglycerate dephosphorylation to form 3-phosphoglycerate and ATP [54]. G6Pase catalyzes the hydrolysis of glucose-6-phosphate to glucose and inorganic phosphate [55]. The lower expression of *PGM*, *PGK1*, *G6Pase*, and *LDH* in *P. vlangalii* from high-altitude populations indicated that *P. vlangalii* decreased glycogen utilization to adapt to the plateau environment, like the Asiatic toad [28,51,52]. The adipogenesis-related genes *FASN* and *SREBF1* and the mitochondrial synthesis of fatty acid gene *MECR* are all important in fatty acid biosynthesis [20,56]. In *P. vlangalii*, the expression of *FASN*, *SREBF1*, and *MECR* in *P. vlangalii* from high-altitude populations indicated the increased storage of fatty acids as a high-altitude adaptation.

FoxO1 plays a crucial role in regulating glucose and lipid metabolism [57,58]; in *FoxO1/3/4* knockout mice, glycolysis increases [59]. FoxO1 could impair glycolysis in various ways [60,61,62], such as by up-regulating *PDK4* expression to affect glucose oxidation [63], and through suppressing MYC signaling to impair glycolysis [62]. In the XDT population of *P. vlangalii*, the expression of *FoxO1* and *PDK4* significantly increased, but the *MYC* expression remained nearly unchanged, which indicated that in lizards, *FoxO1* may up-regulate *PDK4* to reduce the utilization of glycogen in order to adapt to the high altitude. Similar to the related research [57,64], in *P. vlangalii*, up-regulated *FoxO1* increased the transcription of the lipogenesis gene *SREBF1* to enhance the fat activity for high-altitude adaptation. PPAR γ is an important regulator of adipogenesis and lipid metabolism [65]. Endoplasmic reticulum stress induces hepatic steatosis through FoxO1, which stimulated the up-regulation of *PPAR* γ [64]. The activation of the PPAR signaling pathway can promote the proliferation and fat deposition of adipocytes [66]. The overexpression of PPAR γ triggers the expression of adipogenesis-related genes [48,67]. In mice (*Mus musculus*), *PPAR* γ deficiency down-regulates the expression of the target gene *FABP* [68]. In the XDT population, up-regulated *PPAR γ* and activated PPAR signaling pathways may promote fat adipocytes. Therefore, as the altitude increases, the up-regulated expression of *FOXO1* and *PPAR* γ may be a key regulator of high-altitude adaptation.

There are significant differences in metabolism among females differing in reproductive states, as pregnant females have increased metabolic demands [69]. There is a sex-biased pattern to altitude adaptation [70,71]. In high Himalayan frogs (*Nanorana parkeri*), body-size and organ-size variation along elevation gradients show sex-specificity [70]. Immune and neural signaling display sex-specific differences in humans [71]. Additionally, the impact of seasons on the metabolism of heterothermies is also evident [12]. In this study, we only focused on altitude adaptation of male *P. vlangalii* in the active season, which places certain limitations on a more comprehensive understanding of high-altitude adaptation in *P. vlangalii*.

## 5. Conclusions

In conclusion, high-altitude *P. vlangalii* down-regulated the expression of *PGM*, *PCK*, *G6Pase*, and *LDH* to reduce glycogen metabolism, and it up-regulated the expression of *FASN*, along with *SREBF1* to increase fatty acid utilization. Glycogen and fatty acid metabolism change could be triggered by the increasing expression of *FOXO1* and *PPAR γ* in high-altitude *P. vlangalii*. With global warming, animals will shift their distribution toward higher altitudes to seek more opportunities for survival. Due to the limited changes that can be made in gene expression and RMR, it is not easy for *P. vlangalii* to shift its range to higher altitudes. The adaptation mechanisms by which organisms use to adapt to harsh high-altitude environments are very complex, and more efforts should be made in future to draw some general conclusions on this topic.

## Data Availability

The raw sequence reads are available at the China National Center for Bioinformation BIG Submission (https://ngdc.cncb.ac.cn/gsub/; accessed on 23 December 2023) under Bio Project PRJCA023942, the transcriptome data ID CRA015154, and the Metabolome ID OMIX005903-OMIX005909.

## Supplementary Materials

The following supporting information can be downloaded at https://www.mdpi.com/article/10.3390/biology14050459/s1, Figure S1: Trend analysis of gene expression at three altitudes; from left to right are AKS, NCT, and XDT, where the figure up shows the gene number and the figure down shows the *p*-value. Figure S2: GO enrichment analysis of DEGs in NCT vs. AKS, XDT vs. AKS, and XDT vs. NCT. Figure S3: KEGG enrichment analysis of DEGs in NCT vs. AKS, XDT vs. AKS, and XDT vs. NCT. Figure S4: KEGG pathway map linking genes and metabolites in the XDT vs. AKS group, showing the effect of altitude on the glucagon signaling pathway (A) and PPAR signaling pathway (B). Table S1: Size and morphology of male *P. vlangalii* in three populations. Table S2: Genes detected in all samples. Table S3: All differentially expressed genes between any two groups. Table S4: KEGG pathway analysis associated with the grouped DEGs. Table S5: Metabolites detected in all samples. Table S6: All different metabolites between any two groups. Table S7: KEGG pathway analysis associated with the grouped DAMs. Table S8: The gene and metabolite correlation analysis.

## Abbreviations

The following abbreviations are used in this manuscript:

DEGs	Differentially expressed genes
DAMs	Differential metabolites
RMR	Resting metabolic rate
MS	Metabolites
HOAD	β-hydroxyacyl-CoA dehydrogenase
LDH	Lactate dehydrogenase
CS	Citrate synthase
PDK	Pyruvate dehydrogenase kinase

## References

[1] Storz J.F., Moriyama H. (2008). Mechanisms of hemoglobin adaptation to high-altitude hypoxia. High Alt. Med. Biol..

[2] Root T.L., Price J.T., Hall K.R., Schneider S.H., Rosenzweig C., Pounds J.A. (2003). Fingerprints of global warming on wild animals and plants. Nature.

[3] Bertrand R., Lenoir J., Piedallu C., Riofrio-Dillon G., de Ruffray P., Vidal C., Pierrat J.C., Gegout J.C. (2011). Changes in plant community composition lag behind climate warming in lowland forests. Nature.

[4] Zhang K.-L., Yao L.-J., Meng J.-S., Tao J. (2018). Maxent modeling for predicting the potential geographical distribution of two peony species under climate change. Sci. Total Environ..

[5] Storz J.F., Scott G.R., Cheviron Z.A. (2010). Phenotypic plasticity and genetic adaptation to high-altitude hypoxia in vertebrates. J. Exp. Biol..

[6] Ren Y., Liu P.-F., Zhu W.-L., Zhang H., Cai J.-H. (2020). Higher altitude and lower temperature regulate the body mass and energy metabolism in male *Eothenomys miletus*. Pak. J. Zool..

[7] Arias-Reyes C., Soliz J., Joseph V. (2021). Mice and rats display different ventilatory, hematological, and metabolic features of acclimatization to hypoxia. Front. Physiol..

[8] Brummelen A.C.V., Olszewski K.L., Wilinski D., Llinas M., Louw A.I., Birkholtz L.M. (2009). Co-inhibition of *Plasmodium falciparum* S-adenosylmethionine decarboxylase/ornithine decarboxylase reveals perturbation-specific compensatory mechanisms by transcriptome, proteome, and metabolome analyses. J. Biol. Chem..

[9] Liang S.-W., Li W.-X., Zhang Y., Tang X.-L., He J.-Z., Bai Y.-C., Li D.-Q., Wang Y., Chen Q. (2017). Seasonal variation of metabolism in lizard *Phrynocephalus vlangalii* at high-altitude. Comp. Biochem. Physiol. Part A Mol. Integr. Physiol..

[10] Chicco A.J., Le C.H., Gnaiger E., Dreyer H.C., Muyskens J.B., D’Alessandro A., Nemkov T., Hocker A.D., Prenni J.E., Wolfe L.M. (2018). Adaptive remodeling of skeletal muscle energy metabolism in high-altitude hypoxia: Lessons from AltitudeOmics. J. Biol. Chem..

[11] Brandl S.J., Lefcheck J.S., Bates A.E., Rasher D.B., Norin T. (2023). Can metabolic traits explain animal community assembly and functioning?. Biol. Rev..

[12] Toledo L.F., Brito S.P., Milsom W.K., Abe A.S., Andrade D.V. (2008). Effects of season, temperature, and body mass on the standard metabolic rate of tegu lizards (*Tupinambis merianae*). Physiol. Biochem. Zool..

[13] Cheviron Z.A., Bachman G.C., Connaty A.D., McClelland G.B., Storz J.F. (2012). Regulatory changes contribute to the adaptive enhancement of thermogenic capacity in high-altitude deer mice. Proc. Natl. Acad. Sci. USA.

[14] Storz J.F., Cheviron Z.A. (2021). Physiological genomics of adaptation to high-altitude hypoxia. Annu. Rev. Anim. Biosci..

[15] Dawson N.J., Scott G.R. (2022). Adaptive increases in respiratory capacity and O_2_ affinity of subsarcolemmal mitochondria from skeletal muscle of high-altitude deer mice. FASEB J..

[16] Li C., Chen B.-C., Langda S., Pu P., Zhu X.-J., Zhou S.-W., Kalds P., Zhang K., Bhati M., Leonard A. (2024). Multi-omic analyses shed light on the genetic control of high-altitude adaptation in sheep. Genom. Proteom. Bioinform..

[17] Harris P., Castillo Y., Gibson K., Heath D., Arias-Stella J. (1970). Succinic and lactic dehydrogenase activity in myocardial homogenates from animals at high and low altitude. J. Mol. Cell. Cardiol..

[18] Sheafor B.A. (2003). Metabolic enzyme activities across an altitudinal gradient: An examination of pikas (genus *Ochotona*). J. Exp. Biol..

[19] Seebacher F., Sparrow J., Thompson M.B. (2004). Turtles (*Chelodina longicollis*) regulate muscle metabolic enzyme activity in response to seasonal variation in body temperature. J. Comp. Physiol. B.

[20] Qi Y., Zhang T., Wu Y.-Y., Yao Z.-Y., Qiu X., Pu P., Tang X.-L., Fu J.-Z., Yang W.-Z. (2022). A multilevel assessment of plasticity in response to high-altitude environment for Agama lizards. Front. Ecol. Evol..

[21] Kuang L.-D., Zheng Y.-C., Lin Y.-Q., Xu Y.-O., Jin S.-Y., Li Y.-P., Dong F., Jiang Z.-Y. (2010). High-altitude adaptation of yak based on genetic variants and activity of lactate dehydrogenase-1. Biochem. Genet..

[22] Lyons S.A., Tate K.B., Welch K.C., McClelland G.B. (2021). Lipid oxidation during thermogenesis in high-altitude deer mice (*Peromyscus maniculatus*). Am. J. Physiol. Regul. Integr. Comp. Physiol..

[23] Raben N., Nagaraju K., Lee E., Kessler P., Byrne B., Lee L., Lamarca M., King C., Ward J., Sauer B. (1998). Targeted disruption of the acid α-glucosidase gene in mice causes an illness with critical features of both infantile and adult human glycogen storage disease type II. J. Biol. Chem..

[24] Fuson A.L., Cowan D.F., Kanatous S.B., Polasek L.K., Davis R.W. (2003). Adaptations to diving hypoxia in the heart, kidneys and splanchnic organs of harbor seals (*Phoca vitulina*). J. Exp. Biol..

[25] Singh S.N., Vats P., Kumria M.M.L., Ranganathan S., Shyam R., Arora M.P., Jain C.L., Sridharan K. (2001). Effect of high-altitude (7620 m) exposure on glutathione and related metabolism in rats. Eur. J. Appl. Physiol..

[26] Dawson N.J., Alza L., Nandal G., Scott G.R., McCracken K.G. (2020). Convergent changes in muscle metabolism depend on duration of high-altitude ancestry across *Andean waterfowl*. eLife.

[27] Xin J.W., Chai Z.X., Zhang C.F., Yang Y.M., Zhang Q., Zhu Y., Cao H.W., Ji C.D.Y., Zhong J.C., Ji Q.M. (2020). Transcriptome analysis identified long non-coding RNAs involved in the adaption of yak to high-altitude environments. R. Soc. Open Sci..

[28] Li J.-Y., Huang N.-T., Zhang X., Sun C., Chen J.-R., Wei Q. (2024). Changes of collagen content in lung tissues of plateau yak and its mechanism of adaptation to hypoxia. PeerJ.

[29] Yang W.-Z., Qi Y., Fu J.-Z. (2016). Genetic signals of high-altitude adaptation in amphibians: A comparative transcriptome analysis. BMC Genet..

[30] Guo K., Yuan S.-J., Wang H., Zhong J., Wu Y.-Q., Chen W., Hu C.-C., Chang Q. (2021). Species distribution models for predicting the habitat suitability of Chinese fire-bellied newt *Cynops orientalis* under climate change. Ecol. Evol..

[31] Zhang Y., Liang S.-W., He J.-Z., Bai Y.-C., Niu Y.-G., Tang X.-L., Li D.-Q., Chen Q. (2015). Oxidative stress and antioxidant status in a lizard *Phrynocephalus vlangalii* at different altitudes or acclimated to hypoxia. Comp. Biochem. Physiol. Part A Mol. Integr. Physiol..

[32] Li W.-X., Liang S.-W., Wang H.-H., Xin Y., Lu S.-S., Tang X.-L., Chen Q. (2016). The effects of chronic hypoxia on thermoregulation and metabolism in *Phrynocephalus vlangalii*. Asian Herpetol. Res..

[33] Han J.-M., Guo R.-H., Li J.-Q., Guan C., Chen Y., Zhao W. (2016). Organ mass variation in a toad headed lizard *Phrynocephalus vlangalii* in response to hypoxia and low temperature in the Qinghai-Tibet Plateau, China. PLoS ONE.

[34] Zhao E.-M., Adler K. (1993). Herpetology of China.

[35] Yao Y.-T., Du Y., Fang M.-C., Lin L.-H., Ji X. (2019). Developmental stage does not affect resting metabolic rate in the monitor lizard, *Varanus salvator*. Anim. Biol..

[36] Grabherr M.G., Haas B.J., Yassour M., Levin J.Z., Thompson D.A., Amit I., Adiconis X., Fan L., Raychowdhury R., Zeng Q.-D. (2011). Full-length transcriptome assembly from RNA-Seq data without a reference genome. Nat. Biotechnol..

[37] Mortazavi A., Williams B.A., Mccue K., Schaeffer L., Wold B. (2008). Mapping and quantifying mammalian transcriptomes by RNA-Seq. Nat. Methods.

[38] Love M.I., Huber W., Anders S. (2014). Moderated estimation of fold change and dispersion for RNA-seq data with DESeq2. Genome Biol..

[39] Zhang J., Geng X.-Q., Zhang Y.-H., Zhao X.-L., Zhang P.-W., Sun G.-R., Li W.-T., Li D.-H., Han R.-L., Li G.-X. (2022). Interaction between cecal metabolites and liver lipid metabolism pathways during induced molting in laying hens. Front. Physiol..

[40] Smith C.A., Want E.J., O’Maille G., Abagyan R., Siuzdak G. (2006). XCMS: Processing mass spectrometry data for metabolite profiling using nonlinear peak alignment, matching, and identification. Anal. Chem..

[41] Li Q.-F., Sun R.-Y., Huang C.-X., Wang Z.-K., Liu X.-T., Hou J.-J., Liu J.-S., Cai L.-Q., Li N., Zhang S.-Z. (2001). Cold adaptive thermogenesis in small mammals from different geographical zones of China. Comp. Biochem. Physiol. Part A Mol. Integr. Physiol..

[42] Thompson L.J., Downs C.T. (2017). Altitudinal variation in metabolic parameters of a small Afrotropical bird. Comp. Biochem. Physiol. Part A Mol. Integr. Physiol..

[43] Polymeropoulos E.T., Elliott N.G., Frappell P.B. (2017). Hypoxic acclimation leads to metabolic compensation after reoxygenation in Atlantic salmon yolk-sac alevins. Comp. Biochem. Physiol. Part A Mol. Integr. Physiol. A.

[44] Woods A.L., Sharma A.P., Garvican-Lewis L.A., Saunders P.U., Rice A.J., Thompson K.G. (2017). Four weeks of classical altitude training increases resting metabolic rate in highly trained middle-distance runners. Int. J. Sport Nutr. Exerc. Metab..

[45] Plasman M., Bautista A., McCue M.D., De La Vega-Perez A.H. (2020). Resting metabolic rates increase with altitude in a mountain-dwelling lizard. Integr. Zool..

[46] Hodkinson I.D. (2005). Terrestrial insects along elevation gradients: Species and community responses to altitude. Biol. Rev..

[47] Lau D.S., Connaty A.D., Mahalingam S., Wall N., Cheviron Z.A., Storz J.F., Scott G.R., McClelland G.B. (2017). Acclimation to hypoxia increases carbohydrate use during exercise in high-altitude deer mice. Am. J. Physiol. Regul. Integr. Comp. Physiol..

[48] Liu G.-W., Li Y.-H., Liao N., Shang X.-Z., Xu F.-Q., Yin D.-C., Shao D.-Y., Jiang C.-M., Shi J.-L. (2023). Energy metabolic mechanisms for high-altitude sickness: Downregulation of glycolysis and upregulation of the lactic acid/amino acid-pyruvate-TCA pathways and fatty acid oxidation. Sci. Total Environ..

[49] Ding X.-Z., Liang C.-N., Guo X., Wu X.-Y., Wang H.-B., Johnson K.A., Yan P. (2014). Physiological insight into the high-altitude adaptations in domesticated yaks (*Bos grunniens*) along the Qinghai-Tibetan Plateau altitudinal gradient. Livest. Sci..

[50] Ma J., Zhang T.-L., Wang W.-X., Chen Y., Cai W.-T., Zhu B., Xu L.-Y., Gao H.-J., Zhang L.-P., Li J.-Y. (2022). Comparative transcriptome analysis of gayal (*Bos frontalis*), yak (*Bos grunniens*), and cattle (*Bos taurus*) reveal the high-altitude adaptation. Front. Genet..

[51] Yang W.Z., Qi Y., Lu B., Qiao L., Wu Y.Y., Fu J.Z. (2017). Gene expression variations in high-altitude adaptation: A case study of the Asiatic toad (*Bufo gargarizans*). BMC Genet..

[52] Chen Y., Tan S., Fu J.-Z. (2022). Modified metabolism and response to UV radiation: Gene expression variations along an elevational gradient in the Asiatic toad (*Bufo gargarizans*). J. Mol. Evol..

[53] Yomano L.P., Scopes R.K., Ingram L.O. (1993). Cloning, sequencing, and expression of the *Zymomonas mobilis* phosphoglycerate mutase gene (*pgm*) in *Escherichia coli*. J. Bacteriol..

[54] Reddy G.K., Wendisch V.F. (2014). Characterization of 3-phosphoglycerate kinase from *Corynebacterium glutamicum* and its impact on amino acid production. BMC Microbiol..

[55] Wang Y.-D., Oeser J.K., Yang C.-M., Sarkar S., Hackl S.I., Hasty A.H., McGuinness O.P., Paradee W., Hutton J.C., Powell D.R. (2006). Deletion of the gene encoding the ubiquitously expressed glucose-6-phosphatase catalytic subunit-related protein (UGRP)/glucose-6-phosphatase catalytic subunit-β results in lowered plasma cholesterol and elevated glucagon. J. Biol. Chem..

[56] Kim D.-G., Yoo J.-C., Kim E., Lee Y.-S., Yarishkin O.V., Lee D.-Y., Lee K.-H., Hong S.-G., Hwang E.-M., Park J.Y. (2014). A novel cytosolic isoform of mitochondrial trans-2-enoyl-coa reductase enhances peroxisome proliferator-activated receptor α activity. Endocrinol. Metab..

[57] Qu S., Altomonte J., Perdomo G., He J., Fan Y., Kamagate A., Meseck M., Dong H.H. (2006). Aberrant forkhead box O1 function is associated with impaired hepatic metabolism. Endocrinology.

[58] Matsumoto M., Pocai A., Rossetti L., DePinho R.A., Accili D. (2007). Impaired regulation of hepatic glucose production in mice lacking the forkhead transcription factor *FoxO1* in liver. Cell Metab..

[59] Xiong X.-W., Tao R.-Y., DePinho R.A., Dong X.-C. (2013). Deletion of hepatic *FoxO1/3/4* genes in mice significantly impacts on glucose metabolism through downregulation of gluconeogenesis and upregulation of glycolysis. PLoS ONE.

[60] Wilhelm K., Happel K., Eelen G., Schoors S., Oellerich M.F., Lim R., Zimmermann B., Aspalter I.M., Franco C.A., Boettger T. (2016). FOXO1 couples metabolic activity and growth state in the vascular endothelium. Nature.

[61] Yan D., Cai Y., Luo J.-R., Liu J.-J., Li X., Ying F., Xie X., Xu A.-M., Ma X.-S., Xia Z.-Y. (2020). *FOXO1* contributes to diabetic cardiomyopathy via inducing imbalanced oxidative metabolism in type 1 diabetes. J. Cell. Mol. Med..

[62] Wu Y.-L., Guo Y.-Y., Wang Q. (2022). USP21 accelerates the proliferation and glycolysis of esophageal cancer cells by regulating the STAT3/FOXO1 pathway. Tissue Cell.

[63] Piao L., Sidhu V.K., Fang Y.-H., Ryan J.J., Parikh K.S., Hong Z.-G., Toth P.T., Morrow E., Kutty S., Lopaschuk G.D. (2013). FOXO1-mediated upregulation of pyruvate dehydrogenase kinase-4 (PDK4) decreases glucose oxidation and impairs right ventricular function in pulmonary hypertension: Therapeutic benefits of dichloroacetate. J. Mol. Med..

[64] Kim D.H., Ha S., Choi Y.J., Dong H.H., Yu B.P., Chung H.Y. (2019). Altered FoxO1 and PPARγ interaction in age-related ER stress-induced hepatic steatosis. Aging.

[65] Yu S., Matsusue K., Kashireddy P., Cao W.Q., Yeldandi V., Yeldandi A.V., Rao M.S., Gonzalez F.J., Reddy J.K. (2003). Adipocyte-specific gene expression and adipogenic steatosis in the mouse liver due to peroxisome proliferator-activated receptor γ1 (PPARγ1) overexpression. J. Biol. Chem..

[66] Lei M.-M., Li Y.-X., Li J.-Y., Liu J., Dai Z.-C., Chen R., Zhu H.-X. (2024). Low testosterone and high leptin activate PPAR signaling to induce adipogenesis and promote fat deposition in caponized ganders. Int. J. Mol. Sci..

[67] Zhu Y.-P., Jing L., Li X.-Y., Zheng D., Zhou G.-Q., Zhang Y., Sang Y.-J., Shi Z.-X., Sun Z.-W., Zhou X.-Q. (2021). Decabromodiphenyl ether disturbs hepatic glycolipid metabolism by regulating the PI3K/AKT/GLUT4 and mTOR/PPARγ/RXRα pathway in mice and L02 cells. Sci. Total Environ..

[68] Moran-Salvador E., Lopez-Parra M., Garcia Alonso V., Titos E., Martinez-Clemente M., Gonzalez-Periz A., Barak Y., Arroyo V., Claria J. (2011). Role for PPARγ in obesity-induced hepatic steatosis as determined by hepatocyte- and macrophage-specific conditional knockouts. FASEB J..

[69] Angilletta M.J., Winters R.S., Dunham A.E. (2000). Thermal effects on the energetics of lizard embryos: Implications for hatchling phenotypes. Ecology.

[70] Hou D.-M., Jia T., Ren Y., Zhu W.-L., Liu P.-F. (2023). Phenotypic trait variations in the frog *Nanorana parkeri*: Differing adaptive strategies to altitude between sexes. J. Vertebr. Biol..

[71] Nishimura T., Arima H., Koirala S., Ito H., Yamamoto T. (2022). Individual variations and sex differences in hemodynamics and percutaneous arterial oxygen saturation (SpO_2_) in Tibetan highlanders of Tsarang in the Mustang district of Nepal. J. Physiol. Anthropol..

