# Supplementary material for: Transcriptome and Metabolome Analyses Reveal High-Altitude Adaptation in the Qinghai Toad-Headed Lizard Phrynocephalus vlangalii
Source: Biology (Basel). 2025 Apr 24;14(5):459. doi: 10.3390/biology14050459 (PMC12109203; doi:10.3390/biology14050459)
Supplement: Supplementary file 1 [file biology-14-00459-s001.zip › Table S1.docx]

Table S1 Size and morphology of male *P. vlangalii* in three populations.

| Population | NCT | XDT | AKS |
| --- | --- | --- | --- |
| N | 16 | 14 | 13 |
| Body mass (g) | 4.92±0.11  4.08-5.74 | 6.49±0.35  4.33-8.94 | 5.58±0.41  4.14-8.96 |
| Head length (mm) | 10.77±0.17  9.92-11.80 | 10.81±0.19  9.77-11.90 | 11.07±0.23  10.07-12.44 |
| Head width (mm) | 10.20±0.13  9.32-11.33 | 10.51±0.14  9.79-11.30 | 11.16±0.20  10.22-12.19 |
| Head height (mm) | 6.74±0.09  6.15-7.27 | 7.11±0.09  6.46-7.49 | 7.17±0.14  6.28-7.93 |
| Snout-vent length (mm) | 50.11±0.17  49.92-53.25 | 51.67±0.61  47.58-55.09 | 52.66±1.14  46.33-58.20 |
| Abdomen length (mm) | 25.05±0.42  21.73-27.79 | 26.32±0.48  23.52-29.75 | 27.40±1.40  20.10-36.76 |
| Tail length (mm) | 53.03±0.94  45.51-59.50 | 52.92±1.15  48.43-64.49 | 49.40±1.03  45.33-56.45 |
| Fore-limb length (mm) | 15.31±0.27  12.99-17.06 | 15.59±0.23  14.01-17.03 | 15.46±0.39  13.70-17.86 |
| Hind-limb length (mm) | 23.30±0.30  21.05-25.48 | 23.22±0.41  20.46-25.97 | 22.54±0.53  19.56-25.90 |
