# Supplementary figures and images for: Transcriptome and Metabolome Analyses Reveal High-Altitude Adaptation in the Qinghai Toad-Headed Lizard Phrynocephalus vlangalii
Source: Biology (Basel). 2025 Apr 24;14(5):459. doi: 10.3390/biology14050459 (PMC12109203; doi:10.3390/biology14050459)

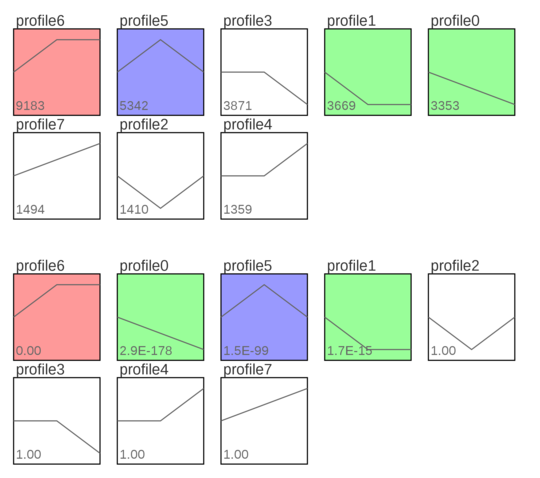

Supplement: Supplementary file 1 [file biology-14-00459-s001.zip › figure S1.tif]

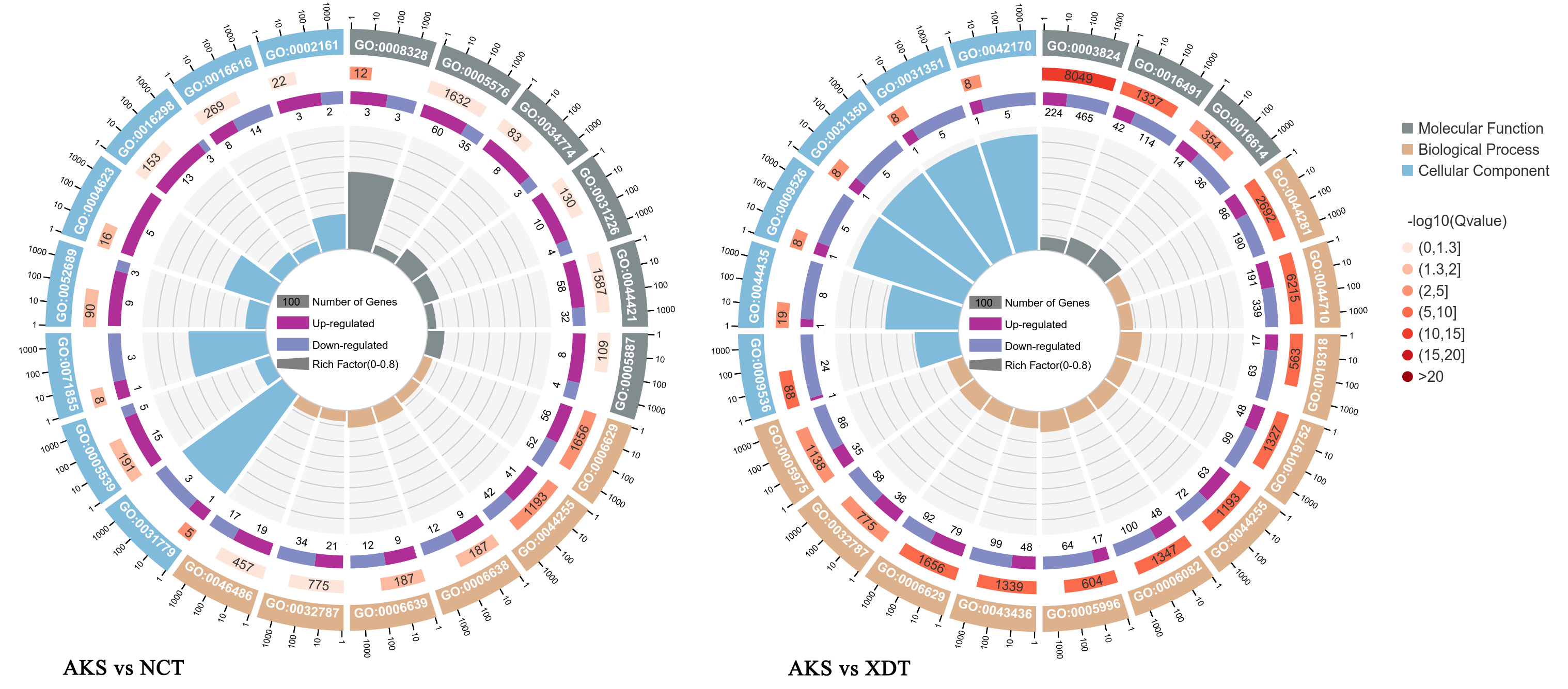

Supplement: Supplementary file 1 [file biology-14-00459-s001.zip › figure S2.tif]

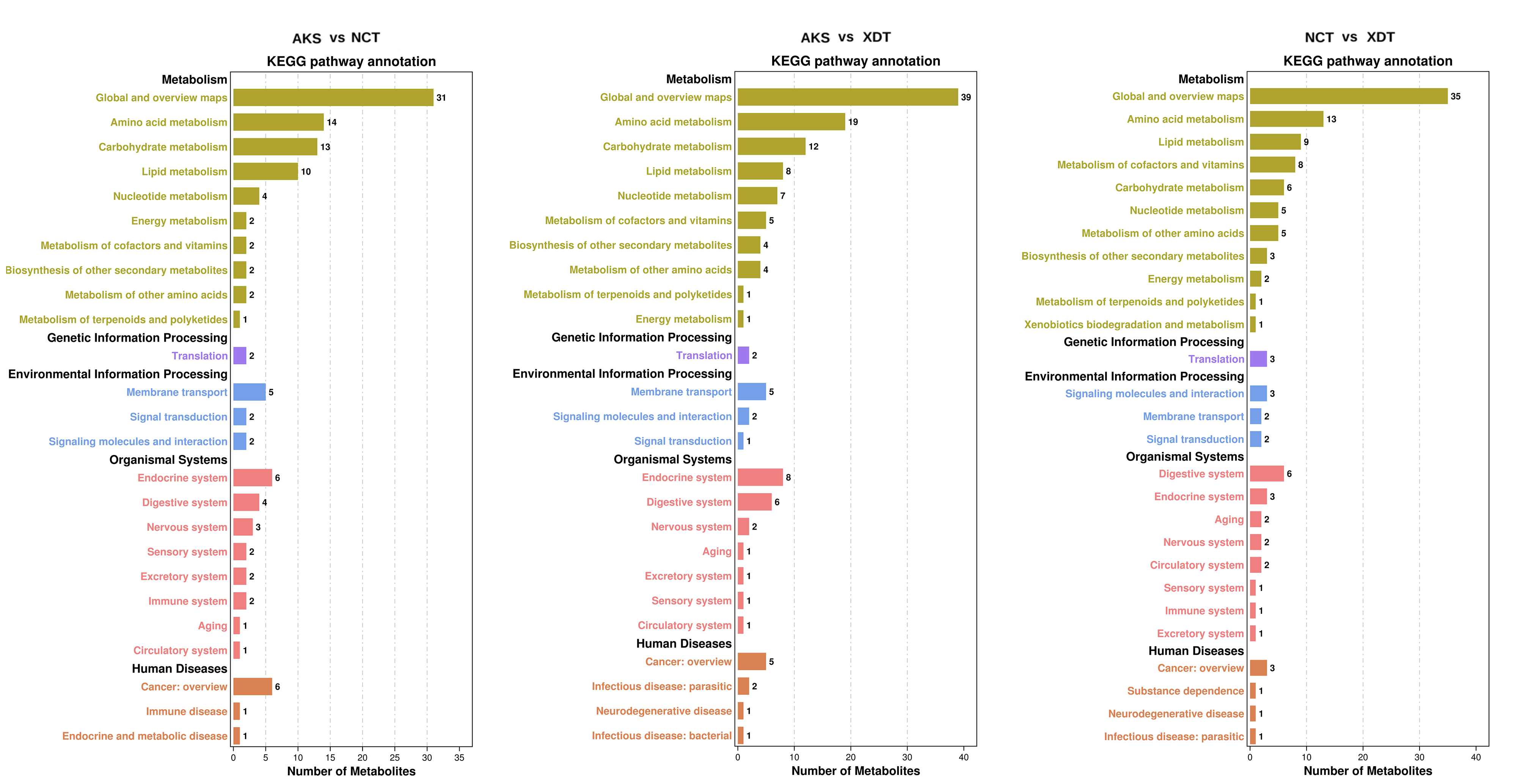

Supplement: Supplementary file 1 [file biology-14-00459-s001.zip › figure S3.tif]
